# Risk adjustment methods for Home Care Quality Indicators (HCQIs) based on the minimum data set for home care

**DOI:** 10.1186/1472-6963-5-7

**Published:** 2005-01-18

**Authors:** Dawn M Dalby, John P Hirdes, Brant E Fries

**Affiliations:** 1 Department of Kinesiology and Physical Education, Wilfrid Laurier University, Waterloo, ON, N2L 3C5, Canada; 2 St. Joseph's Health System Research Network, St. Joseph's Health Centre, Guelph, ON, N1H 5H8, Canada; 3 Department of Health Studies and Gerontology, University of Waterloo, Waterloo, ON, N2L 3G1, Canada; 4 Homewood Research Institute, Homewood Health Centre, 150 Delhi Street, Guelph, ON, N1E 6K9, Canada; 5 Institute of Gerontology, University of Michigan, 300 North Ingalls, Ann Arbor, Michigan, 48109-2007, USA; 6 Ann Arbor VA Medical Center, 2215 Fuller Road, Ann Arbor, Michigan, 48105, USA

## Abstract

**Background:**

There has been increasing interest in enhancing accountability in health care. As such, several methods have been developed to compare the quality of home care services. These comparisons can be problematic if client populations vary across providers and no adjustment is made to account for these differences. The current paper explores the effects of risk adjustment for a set of home care quality indicators (HCQIs) based on the Minimum Data Set for Home Care (MDS-HC).

**Methods:**

A total of 22 home care providers in Ontario and the Winnipeg Regional Health Authority (WRHA) in Manitoba, Canada, gathered data on their clients using the MDS-HC. These assessment data were used to generate HCQIs for each agency and for the two regions. Three types of risk adjustment methods were contrasted: a) client covariates only; b) client covariates plus an "Agency Intake Profile" (AIP) to adjust for ascertainment and selection bias by the agency; and c) client covariates plus the intake Case Mix Index (CMI).

**Results:**

The mean age and gender distribution in the two populations was very similar. Across the 19 risk-adjusted HCQIs, Ontario CCACs had a significantly higher AIP adjustment value for eight HCQIs, indicating a greater propensity to trigger on these quality issues on admission. On average, Ontario had unadjusted rates that were 0.3% higher than the WRHA. Following risk adjustment with the AIP covariate, Ontario rates were, on average, 1.5% *lower *than the WRHA. In the WRHA, individual agencies were likely to experience a decline in their standing, whereby they were more likely to be ranked among the worst performers following risk adjustment. The opposite was true for sites in Ontario.

**Conclusions:**

Risk adjustment is essential when comparing quality of care across providers when home care agencies provide services to populations with different characteristics. While such adjustment had a relatively small effect for the two regions, it did substantially affect the ranking of many individual home care providers.

## Background

In both Canada and the United States efforts are underway to develop systems to assess the quality of health care as a first step to improving services. In the US nursing home sector, the implementation of the Minimum Data Set has been linked to improvements in quality of care [1-4]. Recently, the Centers for Medicare and Medicaid Services (CMS) has developed web pages to give consumers, and the public at large, further information about the quality of nursing homes and home care services across the country. The "Nursing Home Compare" and more recent "Home Health Compare" web sites allow individuals to view several quality indicators (QIs) for individual providers[5,6].

When comparing health care providers across a set of QIs there is a concern that they may differentially admit clients with a greater likelihood of triggering on quality issues. Since the indicators are defined as events that are preferable to avoid (e.g., skin ulcers, untreated pain, weight loss), in the absence of risk adjustment, these providers would appear to be delivering poorer quality of care. Risk adjustment attempts to adjust for different populations of clients who may be at greater risk for experiencing quality issues that is a function of their clinical status rather than the quality of care.

In the US nursing home sector, the CMS recently funded a large-scale project to review all potential long-term care QIs and the risk adjustment process. The evaluation of risk adjustment included a review of both client-level and agency-level covariates. The research team revised the original set of client covariates to create a set of new models and assessed the effects of using an agency-level covariate, the Facility Admission Profile (FAP). The FAP represents the prevalence of the quality issue for residents newly admitted to the facility and was intended to adjust for potential selection bias (i.e., facilities preferentially admitting clients with a greater likelihood of triggering on the indicator) and ascertainment bias (i.e., ability of providers to detect a quality issue that might increase the QI rate). However, after extensive analyses, the research team did not recommend the use of the FAP, given that the FAP did not appear to be a particularly useful measure of ascertainment bias [7].

Nevertheless, in October 2002, the CMS included the FAP in three quality measures reported on the Nursing Home Compare web page[8]. As part of this same initiative, Kidder et al.[9] examined the Nursing Case Mix Index from the RUG-III grouping system as well as seven of the RUG scales as potential risk adjusters. In their final list of quality measures, 12 indicators for chronic care residents and four indicators for post-acute care residents, included a covariate related to the RUG-III system.

### Home Care Quality Indicators (HCQIs)

The current research was part of a project to develop a set of 22 Home Care Quality Indicators (HCQIs) based on items in the Minimum Data Set for Home Care (MDS-HC). Implementation of the MDS-HC is complete or underway in 15 states and in 7 Canadian provinces and territories, where it is being used mainly as a clinical assessment instrument. In Michigan, the MDS-HC is being used as part of the MI-Choice waiver program to reduce nursing home admissions. In Ontario, Community Care Access Centre (CCAC) case managers use the instrument to determine needs, allocate services and make placement decisions.

The HCQI derivation was accomplished using data from Ontario and Michigan since these were the regions with the largest scale implementation in Canada and the US, respectively. In their paper describing the HCQI derivation, Hirdes et al.[10] recommended the use of client-level risk adjustment for all but four indicators, and suggested the use of the Agency Intake Profile (AIP) as another method for risk adjustment. These various approaches to risk adjustment are the focus of this study.

The set of HCQIs used in this study were developed by members of inter*RAI*, a non-profit multinational organization dedicated to the development and refinement of assessment instruments for older adults and persons with disabilities, and their related applications. The HCQIs represent outcome measures that document an agency's rate for triggering on a quality issue. For example, one indicator measures the prevalence of weight loss among individuals who are not considered to be palliative clients. The HCQIs include a mix of prevalence measures (i.e., measured on a cross-section of clients at one point in time) and incidence measures (i.e., failure to improve or incidence of an event measured across two points in time). All HCQIs are defined as events to be avoided such that a higher rate on the indicator is indicative of poorer performance.

This paper uses a dataset from two Canadian provinces to further explore these three types of risk adjustment for the inter*RAI *HCQIs. It explores the effects of risk adjustment at both the level of the region and at the level of the individual home care provider.

## Methods

### Data

The RAI Health Informatics Project was a two and a half year research study begun in 1999 in which fourteen of Ontario's 43 CCACs used the MDS-HC as part of usual practice for all adult (18 years and older) home care clients. CCACs provide several different services: information and referral, case management and placement in long-term care facilities. CCACs purchase in-home services from external contracted agencies through a "request for proposal" process. Services provided include in-home physiotherapy, occupational therapy, personal support and homemaking, medical supplies and equipment, and care by other professional such as social workers, dietitians and speech-language pathologists. Case managers oversee the assessment process, make referrals to service providers and then monitor the care provided in the home. Funding for CCACs is based on an annual budget provided by the Ontario Ministry of Health and Long-term Care. CCACs are governed by independent, non-profit boards of directors, accountable to the Ministry of Health and Long-term Care.

In Ontario, it is now mandatory for all long stay home care clients to be assessed with the MDS-HC. However, at the time of the RAI Health Informatics project, the MDS-HC was not mandated for use. As such, some clients (e.g., those expected to be on service less than two weeks) did not receive an MDS-HC assessment and were not included in the current project.

At this same time, Manitoba conducted a pilot implementation of the MDS-HC in the fourteen offices of the Winnipeg Regional Health Authority (WRHA). The WRHA is one of twelve health authorities in the province, and is responsible for providing health services to the approximately 646,000 residents of Winnipeg and the surrounding suburban area. The WRHA receives annual funding from the government of Manitoba. The home care program of the WRHA was established in 1974 with a mandate to provide effective and responsive health care services in the community to support independent living and facilitate admission into LTC facilities when independent community living is no longer an option. Home care services include personal care, nursing, counselling, occupational therapy and physiotherapy assessment, referral to other agencies and coordination of services.

In the WRHA region, an MDS-HC assessment was completed by care coordinators only if the client was anticipated to be on service for at least 90 days. As a result, the study sample was focused on a long stay population of home care clients.

All participating sites in the WRHA and in Ontario identified a set of case managers who received a two-day training session led by a member of the research team. They then used the MDS-HC instrument as part of their usual in-home assessment.

The data used for this study represent a cross-sectional cohort of home care clients assessed between November, 1999 and December, 2002. Two CCACs in Ontario and four WRHA sites, were removed from the database because they submitted fewer than 20 assessments, resulting in a total of ten WHRA and twelve Ontario sites. Of these, three Ontario CCACs and eight offices of the WRHA also submitted client reassessments at approximately 90 days, which allowed for the calculation of failure to improve/incidence HCQIs.

The protocol for data collection was reviewed and received ethics clearance through the Office of Research Ethics at the University of Waterloo, Canada.

### Risk adjustment

Risk adjustment attempts to adjust for differences in client populations that may bias the HCQI rates. Organizations that provide care to more impaired clients will tend to have higher unadjusted rates, regardless of the quality of care they provide. As such, risk adjustment methods are used to maximize the ability to make fair comparisons across providers. With any type of risk adjustment, caution must be exercised to prevent over adjustment (i.e., adjusting away poor practice). The choice of risk adjustment covariates is therefore highly important and should not include variables that would be considered to reflect suboptimal clinical care.

There are two generic types of risk adjustment that have been recommended for the HCQIs. The first adjusts for differences in the population at the client-level[10]. Potential adjusters can include both individual assessment items and summary scales embedded within the MDS-HC. The HCQI developers evaluated a large range of potential covariates, considering their distributional properties, strengths of association with the outcomes of interest, consistency of findings across jurisdictions and potential for clinically inappropriate adjustment (e.g., benzodiazepine use was not considered a reasonable adjuster for falls). As a result, from zero to five risk adjusters were recommended for the 22 HCQIs and these client covariates were used in the current project[11].

The second type of risk adjustment was intended to control for two types of bias at the agency level: a) the agency's ability to identify differences in clients' clinical characteristics and b) differences in who an agency selects for admission. These risk adjustments are performed at the agency level after the individual-level risk adjustments are applied.

The Agency Intake Profile (AIP) was used following the methodology outlined by Morris et al., for the MDS 2.0 quality indicators[7]. The AIP was calculated for each agency based on clients for whom the MDS-HC assessment was their intake assessment or for clients who had been on service for no more than 30 days. This group of clients was considered to be the intake cohort.

More recently, an alternative form of agency-level risk adjustment has been proposed, to control for potential selection and ascertainment bias. This adjustment employs the Case Mix Index (CMI) associated with the RUG-III/HC methodology[9]. In this instance, the CMI for the intake cohort was calculated, and adjustments to the HCQIs were performed similar to those used for the AIP.

The process followed for adjusting HCQI rates was the same as that used by Morris et al. in adjusting the US nursing home QI rates[7]. Three sources of information are required: the agency-level observed rate, the agency-level expected rate and the grand mean across all agencies. The first data element was simply the raw observed HCQI rate for a given home care agency. The next data element involved the creation of an expected rate for each client within a given agency, based on output from a logistic regression model. In this model, each HCQI acts as a dichotomous dependent variable (i.e., triggering on the HCQI or not) and the client-level and/or agency-level covariates are entered simultaneously as independent variables. The expected value for each client is then pooled to create an expected value for the agency. Three separate risk adjusted HCQI rates are thus computed, based on which method is used for adjustment: the expected rate based on the client covariates only (CC), on client covariates plus AIP (+AIP) and on client covariates plus CMI (+CMI).

The final adjusted value can be thought of as an estimate of an agency's HCQI rate if the agency had clients with an average level of risk[7]. This risk adjustment method is similar to the concept of indirect standardization, in which the ratio of the observed to expected events is calculated then multiplied by the crude rate in the standard population[12]. In the current project, the standard population used was the combined set of agencies from both the Winnipeg Regional Health Authority (WRHA) and from Ontario.

### HCQI rates

For each participating agency, the unadjusted HCQI rates were calculated for all 22 indicators. These rates represent the average rate across all eligible clients for a given agency. For prevalence indicators, the rates were calculated only for clients who had been on service for at least 30 days to avoid penalizing an organization for quality issues that were newly recognized.

Several methods were utilized to assess the impact of risk adjustment, both at the regional and at the agency-level. The unadjusted and three adjusted rates were compared between Ontario and the WRHA, to assess the effects at the regional level. At the level of the individual agency, ranks were calculated for each HCQI and a count was created to examine how often a given agency was among those with the four highest rates. Since higher rates on each HCQI were indicative of higher prevalence or incidence of undesirable outcomes, agencies ranked within the four highest rates were considered to be among the "worst performers." The range between agencies with the highest and fourth highest rates was also examined for both unadjusted and adjusted rates to assess the degree of variation among the group of worst performers.

## Results

The two regions were very similar on average age and sex. The WRHA clients were significantly less likely to have some level of cognitive impairment, as measured by the Cognitive Performance Scale (CPS)[13], compared to Ontario clients, although the actual difference was only 2.6% (WRHA: 37.0% vs. Ontario: 39.6%; p < 0.0001). They were also significantly less likely than clients in Ontario to require some assistance with ADLs (22.0% vs. 27.1%, respectively; p < 0.0001), as measured by the ADL Self-performance Hierarchy Scale[14]. Severe daily pain was experienced by 17.2% of the Ontario clients compared to 14.2% of the WRHA clients (p < 0.0001). Although the differences were statistically significant, with Ontario having significantly lower rates of both arthritis and hypertension, the absolute difference between the regions on the most common diagnoses was less than 5% (Table 1).

**Table 1:** Characteristics of home care clients in the WRHA and Ontario*

	**WRHA (n = 6704)**	**ON (n = 5063)**	**p value**
	**%**	**%**	

**Age**			
Mean (95% CI**)	76.0 (74.9, 77.0)	75.6 (75.2, 76.0)	0.49
18–64	13.4	16.5	<0.0001
65–74	16.5	19.2	
75–84	39.9	40.7	
85 and older	30.0	23.6	
**Sex**			
Female	69.2	70.5	0.14
**Marital status**			
Never married	11.5	8.7	<0.0001
Married/widowed	79.4	82.7	
Separated/divorced	8.2	7.9	
Other	0.8	0.7	
**Primary language**			
English	85.6	85.1	0.63
French	3.5	3.8	
Other	10.9	11.1	
**Aboriginal status**			
Origin is Inuit, Metis or North American Indian	3.2	1.6	<0.0001
**Cognitive Performance Scale**			
Mean (95% CI)	0.8 (0.8, 0.8)	0.8 (0.8, 0.9)	0.07
0 – intact	63.0	60.4	<0.0001
1 – borderline intact	15.0	18.4	
1 – borderline intact	15.0	18.4	
2 – mild impairment	8.4	7.3	
3 – moderate impairment	10.9	10.1	
4 – moderate to severe impairment	0.8	1.0	
5 – severe impairment	1.5	2.5	
6 – very severe impairment	0.5	0.4	
**ADL Self-Performance Hierarchy Scale**			
Mean (95% CI)	0.5 (0.5, 0.6)	0.7 (0.6, 0.7)	<0.0001
0 – independent	78.0	72.9	<0.0001
1 – supervision required	5.2	7.9	
2 – limited impairment	8.9	8.5	
3 – extensive assistance required (level I)	4.7	5.4	
4 – extensive assistance required (level II)	1.6	2.6	
5 – dependent	1.1	2.0	
6 – total dependence	0.5	0.7	
**Pain Scale**			
Mean (95% CI)	1.2 (1.2, 1.2)	1.3 (1.3, 1.4)	<0.0001
0 – no pain	39.8	34.9	<0.0001
1 – less than daily pain	14.4	14.1	
2 – daily pain but not severe	31.6	33.8	
3 – severe daily pain	14.2	17.2	
Top 3 medical diagnoses***			
Arthritis	49.4	44.8	<0.0001
Hypertension	42.2	37.4	<0.0001
Diabetes	19.1	20.1	0.24

* client characteristics and scale values based on first submitted MDS-HC assessment

** CI = confidence interval

*** disease was present and was or was not being treated or monitored by a home care professional

### Unadjusted rates

When comparing the two regions, there were statistically significant differences for five of the 22 unadjusted HCQIs (Table 2). In only one of these five cases (ADL rehabilitation potential and no therapies) was the rate in the WRHA significantly higher than the rate in Ontario. However, the actual size of the absolute difference between regions was small (0.3% on average). Among the prevalence HCQIs, the largest unadjusted difference was for disruptive/intense daily pain, which was 9.1% higher in Ontario. There were no statistically significant differences between the regions for any of the incidence HCQIs.

**Table 2:** Unadjusted HCQI rates comparing the WRHA and Ontario*

	**WRHA Offices**		**Ontario CCACs**		p value**
	**n = 10**		**n = 12**		

***Prevalence HCQIs***	**Mean (sd)**	**95% CI**	**Mean (sd)**	**95% CI**	

Inadequate meals	2.7 (1.2)	1.9, 3.6	4.4 (1.9)	2.1, 7.1	0.02
Weight loss	5.7 (3.4)	3.2, 8.1	7.6 (2.2)	5.1, 10.3	0.13
Dehydration	0.6(0.7)	0.08, 1.1	2.4 (1.6)	1.0, 5.0	0.003
Not receiving a medication review by a physician	3.8 (1.8)	2.5, 5.0	10.3 (4.3)	6.6, 17.1	0.0002
No assistive device among clients with difficulty in locomotion	13.8 (16.4)	2.1, 25.5	10.2 (5.2)	4.2, 15.6	0.52
ADL/rehabilitation potential and no therapies	84.0 (7.4)	78.7, 89.3	73.7 (5.5)	69.6, 78.5	0.001
Falls	20.8 (7.8)	15.2, 26.3	24.4 (4.5)	19.7, 30.3	0.18
Social isolation	21.6 (4.5)	18.4, 24.8	24.3 (5.9)	17.6, 32.4	0.24
Delirium	5.1 (2.3)	3.4, 6.7	5.9 (2.6)	3.4, 9.0	0.45
Negative mood	7.9 (3.2)	5.6, 10.1	11.6 (6.2)	5.2, 18.4	0.10
Disruptive or intense daily pain	30.5 (4.0)	27.6, 33.3	39.6 (6.3)	33.6, 44.3	0.0008
Inadequate pain control among those with pain	26.6 (5.3)	22.8, 30.4	29.7 (7.4)	22.3, 35.8	0.27
Neglect/abuse	3.0 (2.6)	1.2, 4.9	2.5 (2.1)	0.8, 5.2	0.58
Injuries	13.6 (6.4)	9.0, 18.2	11.0 (3.7)	8.6, 14.3	0.26
No influenza vaccine	28.4 (7.1)	23.2, 33.5	30.3 (9.8)	21.1, 44.0	0.61
Hospitalization	32.0 (7.2)	26.8, 37.2	36.4 (4.9)	31.1, 40.4	0.11

***Incidence HCQIs***	**n = 8**		**n = 3**		

Failure to improve/incidence of bladder incontinence	29.5 (9.7)	21.4, 37.5	23.1 (7.4)	4.8, 41.4	0.33
Failure to improve/incidence of skin ulcers	3.4 (4.54)	0.0, 7.1	3.4 (3.2)	-5.0, 11.3	0.96
Failure to improve/incidence of decline on ADL long form	41.5 (12.5)	31.1, 51.9	30.3 (4.4)	19.3, 41.3	0.17
Failure to improve/incidence of impaired locomotion in the home	11.0 (9.0)	3.5, 18.5	16.5 (9.2)	-6.5, 38.4	0.44
Failure to improve/incidence of cognitive decline	44.6 (13.7)	33.1, 56.0	36.1 (3.9)	26.3, 45.9	0.33
Failure to improve/incidence of difficulty in communication	13.7 (8.1)	6.9, 20.4	14.4 (3.4)	6.0, 22.9	0.88

* in the event that the lower value of the 95% confidence interval was less than zero, the value was set to zero.

** based on a t-test of independent means.

### Agency-level covariates

The AIP was calculated for each home care agency for each of the 19 HCQIs for which client-level risk adjustment was recommended. The AIP values shown represent the HCQI rate among an admission cohort within each region and were used in the risk adjustment models that included client-level covariates together with the AIP covariate. Ontario CCACs had a significantly higher AIP value for eight indicators, demonstrating that they admit or at least assess individuals as more likely to have these conditions on admission (Table 3).

**Table 3:** AIP values for the WRHA and Ontario for each of the 19 risk-adjusted HCQIs*

	**WRHA n = 10**	**Ontario n = 12**	**p value**
***Prevalence HCQIs***	**mean (95% CI)**		

Inadequate meals	2.9 (1.7, 4.1)	6.8 (5.3, 8.2)	0.0003
Weight loss	8.2 (4.7, 11.7)	12.8 (10.7, 14.8)	0.02
Dehydration	0.6 (0.2, 1.1)	4.0 (2.5, 5.5)	0.0005
No assistive device among clients with difficulty in locomotion	13.4 (10.8, 16.0)	19.1 (12.7, 25.4)	0.09
Falls	24.4 (20.6, 28.2)	31.4 (27.5, 35.3)	0.01
Social isolation	22.4 (19.4, 25.3)	25.3 (21.2, 29.4)	0.23
Delirium	5.2 (4.0, 6.4)	10.2 (7.8, 12.6)	0.0008
Negative mood	7.7 (5.5, 9.9)	12.8 (8.7, 17.0)	0.03
Disruptive or intense daily pain	31.5 (28.1, 34.9)	42.3 (37.5, 47.0)	0.0008
Inadequate pain control among those with pain	30.6 (26.6, 34.6)	31.4 (26.5, 36.4)	0.79
Neglect/abuse	2.5 (1.5, 3.5)	2.9 (1.3, 4.6)	0.65
Injuries	11.5 (8.5, 14.5)	12.8 (9.1, 16.5)	0.55
Hospitalization	35.3 (31.6, 39.1)	52.0 (47.4, 56.6)	<0.0001

***Incidence HCQIs***	**n = 8**	**n = 3**	

Failure to improve/incidence of bladder incontinence	0.9 (0.8, 1.0)	0.9 (0.5, 1.2)	0.81
Failure to improve/incidence of skin ulcers	6.3 (5.6, 7.0)	7.6 (4.8, 10.3)	0.07
Failure to improve/incidence of decline on ADL long form	1.9 (1.6, 2.3)	2.8 (0.0, 6.9)	0.45
Failure to improve/incidence of impaired locomotion in the home	0.3 (0.2, 0.4)	0.6 (0.0, 1.6)	0.36
Failure to improve/incidence of cognitive decline	0.8 (0.7, 0.9)	1.0 (0.2, 1.7)	0.21
Failure to improve/incidence of difficulty in communication	0.5 (0.4, 0.6)	0.6 (0.0, 1.6)	0.65

* for prevalence HCQIs and failure to improve/incidence of skin ulcers, the AIP value represents the mean rate (expressed as a percent) of the HCQI on an intake cohort of clients and for the remaining incidence HCQIs, the AIP value represents the mean value for the MDS item(s) involved in calculating the HCQI.

Among new home care clients in Ontario, 52% of clients triggered on the HCQI for the prevalence of hospitalization versus 35% in the WRHA (difference of 17%).

Ontario also had a significantly (p < 0.0001) higher Case Mix Index (CMI) on intake (i.e., the CMI covariate), at 0.84 (95% CI: 0.81, 0.86), compared with the WRHA at 0.70 (CI: 0.68, 0.71).

### Risk adjustment at the regional level

In general, the risk adjustment process minimized the differences between the two regions compared with the unadjusted rates. For example, the unadjusted difference between regions for the prevalence of disruptive/intense daily pain was 9.1%, however, the difference was reduced to 8.0% with the CC adjustment, 5.7% with the +CMI adjustment and 2.8% with the +AIP adjustment (Table 4). The +AIP adjusted rates showed the most variability, so that for five HCQIs, the direction of the difference was reversed. For example, the unadjusted rate of falls was 3.6% ***higher ***in Ontario than in the WRHA. Following the +AIP adjustment, Ontario had a rate that was 1.4% *lower *than in the WRHA.

**Table 4:** Difference between Ontario and WRHA HCQI unadjusted and adjusted rates*

	**Unadjusted**	**Client covariates only**	**AIP + client covariates**	**CMI+ client Covariates**
	**Difference (ON-WRHA) expressed as a %**			

***Prevalence HCQIs***				

Inadequate meals	1.7	1.7	0.2	1.5
Weight loss	1.9	1.9	0.2	1.5
Dehydration	1.8	1.9	1.1	1.7
No assistive device among clients with difficulty in locomotion	-3.6	-1.9	-2.8	-3.3
Falls	3.6	1.7	-1.4	0.7
Social isolation	2.7	0.8	1.1	0.6
Delirium	0.8	1.1	-0.4	0.8
Negative mood	3.7	3.1	-1.0	1.8
Disruptive or intense daily pain	9.1	8.0	2.8	5.7
Inadequate pain control among those with pain	3.1	2.8	1.7	3.3
Neglect/abuse	-0.5	-0.4	-0.5	-1.0
Injuries	-2.6	-3.0	-3.3	-1.8
Hospitalization	4.3	2.8	-1.4	2.1

***Incidence HCQIs***				

Failure to improve/incidence of bladder incontinence	-6.4	-5.7	-5.6	-2.3
Failure to improve/incidence of skin ulcers	0.0	0.0	-1.2	-0.8
Failure to improve/incidence of decline on ADL long form	-11.2	-11.0	-10.7	-6.6
Failure to improve/incidence of impaired locomotion in the home	5.0	5.3	-0.1	4.2
Failure to improve/incidence of cognitive decline	-8.5	-6.1	-7.7	-4.1
Failure to improve/incidence of difficulty in communication	0.7	1.3	0.0	1.7
***Mean***	***0.3***	***0.2***	***-1.5***	***0.3***

* differences were always calculated as Ontario rate minus WRHA rate

### Agencies with the highest rates

A summary measure was created to count the frequency with which an agency was ranked among the worst performers. Overall, only three of the ten offices within the WRHA did not change their ranking when rates were adjusted (Table 5). For example, Office 6 was ranked within the four highest (i.e., worst) agencies for three out of the 19 HCQIs both for unadjusted and for the three adjusted rates. In another six offices, the effect of risk adjustment was a decline in their standing (i.e., poorer quality) so that after at least one type of risk adjustment they were ranked among the worst performers. For example, in Offices 2, 3 and 7, the number of times they were ranked within the four highest rates increased as a result of the +AIP adjustment. The most dramatic effect was evident in Office 7 such that the unadjusted rates ranked this group among the worst performers only once, but the +AIP adjustment increased this frequency to five. There was no instance of an office in the WRHA consistently benefiting from risk adjustment.

**Table 5:** Number of times a given agency was ranked within the four highest rates

	**Number of times agency ranked within the four highest rates**			
	**Unadjusted**	**Client covariates**	**AIP + client covariates**	**CMI + client covariates**

**WRHA**				

1	8	9	10	9
2	1	1	4	3
3	2	2	5	3
4*	0	0	0	0
5	3	5	4	5
6	3	3	3	3
7	1	2	5	2
8	0	0	2	0
9	7	6	8	6
10*	4	4	4	4

**Ontario**				

100*	3	3	2	4
200	5	5	4	4
300*	1	1	2	2
400*	5	5	6	4
500*	1	2	0	2
600*	4	3	3	1
700*	1	1	2	1
800*	5	4	2	4
900*	6	6	6	6
1000*	1	1	0	1
1100	10	9	3	8
1200	6	4	1	4

* indicates agencies for which the count was based on 13 HCQIs since incidence HCQIs could not be calculated

In Ontario, only CCAC 900 experienced no change in their ranking following risk adjustment (Table 5). In another nine cases, at least one type of adjustment resulted in an improved standing, with the agency ranking among the four highest rates less often. For example, CCAC 1100 ranked among the worst performers ten times across the 19 HCQIs prior to risk adjustment, but the +AIP adjustment reduced this value to three. A similar effect was observed for CCAC 1200 which was ranked in the worst four performers six times across the unadjusted rates, but only once following +AIP adjustment.

Examining only the ranking of agencies may be misleading if very small changes in the actual rate resulted in an increase or decrease in the rank for a given agency. For example, the change in the rate for a given agency could be very small, say 5%, but could result in the organization moving from the fifth highest rate (i.e., fifth worst performer) to the third highest rate. Examining only the ranking tells one little about the actual magnitude of change among the worst performers. Therefore, it is also important to explore the range in the rates across the four highest agencies.

The unadjusted and CC adjusted rates had the largest degree of variation between the highest and fourth highest agency, with a mean of 11% across the 19 HCQIs. The +CMI adjustment had the next largest amount of variation at 10% and the +AIP adjustment at 8% (Table 6). These results further reinforce the finding that the +AIP method of adjustment consistently had the largest impact compared with the other types of risk adjustment.

**Table 6:** Range in the HCQI rates comparing agencies with the highest rates

	**Unadjusted**		**Client covariates**		**AIP + client covariates**		**CMI + client covariates**	
	**Range between 1^st ^and 4^th ^highest agency**	**Difference between 1^st ^and 4^th ^agency**	**Range between 1^st ^and 4^th ^highest agency**	**Difference between 1^st ^and 4^th ^agency**	**Range between 1^st ^and 4^th ^highest agency**	**Difference between 1^st ^and 4^th ^agency**	**Range between 1^st ^and 4^th ^highest agency**	**Difference between 1^st ^and 4^th ^agency**

	**%**		**%**		**%**		**%**	

***Prevalence HCQIs***								

Inadequate meals	7.4 – 4.9	2.5	8.3 – 5.4	2.9	6.4 – 4.6	1.8	8.2 – 5.4	2.9
Weight loss	13.3 – 9.3	4.0	13.3 – 9.2	4.1	10.7 – 9.2	1.5	13.6 – 9.8	3.8
Dehydration	6.1 – 2.6	3.5	6.6 – 3.0	3.6	3.8 – 2.3	1.5	6.5 – 2.8	3.7
No assistive device among clients with difficulty in locomotion	60.0 – 15.3	44.7	50.4 – 14.8	35.6	53.8 – 15.6	38.2	52.0 – 14.3	37.7
Falls	38.4 – 28.8	9.6	38.4 – 28.8	9.6	36.5 – 28.4	8.1	39.1 – 29.1	10.0
Social isolation	33.1 – 28.6	4.5	31.8 – 30.2	1.6	31.5 – 30.3	1.2	31.9 – 30.2	1.7
Delirium	11.0 – 8.1	2.9	14.3 – 12.4	1.9	12.8 – 11.9	0.9	14.2 – 12.9	1.3
Negative mood	26.2 – 13.4	12.8	30.4 – 16.1	14.3	18.0 – 15.0	3.0	23.4 – 15.9	7.5
Disruptive or intense daily pain	54.1 – 42.5	11.6	51.6 – 40.8	10.8	43.7 – 39.4	4.3	44.5 – 41.2	3.3
Inadequate pain control among those with pain	46.5 – 32.6	13.9	47.6 – 33.8	13.8	36.6 – 33.0	3.6	47.4 – 34.2	13.2
Neglect/abuse	9.7 – 3.9	5.8	9.5 – 4.0	5.5	6.7 – 3.7	3.0	9.8 – 4.4	5.4
Injuries	22.4 – 18.9	3.5	22.8 – 18.3	4.5	21.9 – 16.1	5.8	22.0 – 19.0	3.0
Hospitalization	46.8 – 40.1	6.7	44.6 – 40.8	3.8	47.7 – 38.4	9.3	46.2 – 41.2	5.0

***Incidence HCQIs***								

Failure to improve/incidence of bladder incontinence	48.0 – 29.0	19.0	48.4 – 28.4	20.0	44.5 – 31.9	12.6	47.3 – 27.9	19.4
Failure to improve/incidence of skin ulcers	12.9 – 4.2	8.7	13.2 – 4.3	8.9	11.1 – 5.5	5.6	14.1 – 4.9	9.2
Failure to improve/incidence of decline on ADL long form	56.0 – 42.9	13.1	55.1 – 43.4	11.7	54.9 – 43.3	11.6	53.9 – 41.2	12.7
Failure to improve/incidence of impaired locomotion in the home	29.0 – 14.1	14.9	33.4 – 19.1	14.3	36.6 – 22.2	14.4	34.1 – 18.4	15.7
Failure to improve/incidence of cognitive decline	72.0 – 48.4	23.6	70.1 – 46.7	23.4	69.5 – 46.3	23.2	69.6 – 46.0	23.6
Failure to improve/incidence of difficulty in communication	23.8 – 18.3	5.5	30.7 – 22.3	8.4	29.3 – 22.4	6.9	30.6 – 22.4	8.2
**Mean difference**	**11.1**		**10.5**		**8.2**		**9.9**	

## Discussion

Ontario CCACs exhibited several key differences when compared to the WRHA. Ontario sites had significantly higher unadjusted rates across four HCQIs. However, the degree of variation between the sites was small, with a mean difference across indicators of less than 1%. Ontario as a region had significantly higher AIP values for eight HCQIs, indicating a greater propensity in Ontario to admit clients exhibiting quality issues, and they also had a higher mean Case Mix Index.

Overall, the change in the actual rates at the regional level was small following risk adjustment. When risk adjustment was applied, for the client covariates alone or the client covariates with the CMI covariate, there was little effect on the mean difference between the two regions. In each case, the mean difference was positive (i.e., Ontario had a higher rate on average) and less than 1%. The +AIP adjustment, however, resulted in a negative mean difference (i.e., WRHA higher than Ontario on average) across the set of risk-adjusted HCQIs.

At the agency level, there was a greater influence of risk adjustment, as assessed by agency rankings across the set of indicators. In general, sites in Ontario benefited from risk adjustment and were less likely to be ranked among the worst performers. The opposite was true within the WRHA. Again, the +AIP adjustment had the largest influence when compared to the other two types of risk adjustment.

It is possible that the level of variation between agencies on the AIP covariate was greater than the level of variation between clients on the individual-level covariates. Although a detailed analysis is beyond the scope of this paper, use of a single HCQI example may prove beneficial. When examining the prevalence of falls (an HCQI that showed the largest changes in the agency rankings following risk adjustment), the coefficient of variation (CV) for the AIP value was 23.0. The CV is a relative measure of dispersion about the mean and is calculated by dividing the standard deviation by the mean and multiplying by 100. This CV value was much lower than any of the corresponding CV values for the various MDS outcome scales that reflect differences at the level of the individual client. For example, the CV for the Cognitive Performance Scale was 158.5 and for the ADL Self-performance Hierarchy Scale, the CV was 195.6 (data not shown).

Clearly, the +AIP adjustment had the effect of minimizing differences between the individual providers and it also resulted in rates that had less variability, as demonstrated by the drop in the CV value when comparing the unadjusted and +AIP adjusted rates for the prevalence of falls indicator (CV of 27.7 and 19.1, respectively; data not shown). However, this effect cannot be explained by differences in the variances of the adjusters since the AIP had a lower level of variance than the individual covariates.

It is also possible that the AIP covariate serves as a proxy for regional differences in practice patterns. If the Ontario agencies are grouped into four main geographic regions, the AIP value for the falls HCQI ranged from 24.1% to 34.0%. The degree of variation appears modest, but cannot be discounted as one factor in the ability of the AIP adjustment to minimize differences between providers and between regions.

Although there continues to be support for the conceptual notion of risk adjustment, the potential to over adjust remains a concern. In a recent publication, Mor et al[15] recognize the possibility for over adjustment and conclude that in the absence of a simple solution to this problem, researchers must carefully consider each performance measure on an individual basis in an attempt to minimize this issue.

In the current project, the AIP covariate represents a continuous, numeric value that can range from zero to one. Thus, it does not simply serve as an agency identifier, but represents the rate among an intake cohort. Furthermore, in the US, Morris et al. determined that the Facility Admission Profile, the conceptual cousin to the AIP, did not substantially improve the risk adjustment models and they did not recommend its use. This decision was not based on a fear of over adjustment, but rather a concern that the FAP added increased complexity without significantly improving the risk adjustment process[7].

It is also important that to develop a clearer understanding of what is meant by "over adjustment". For example, it would seem reasonable to differentiate between instances of truly *inappropriate *risk adjustment where variance is due to poor practices (e.g., adjusted for benzodiazepine use for a falls indicator) and "over adjustment" due to the excessive use of spurious risk adjusters resulting in suppression of variance. Another possible example of over adjustment is the use of individual level covariates that are too closely related to the quality indicator. For example, one might argue that using dressing of the upper body as a risk adjuster for a QI on dressing the lower body is a form of over adjustment because the dependent variable is represented on both sides of the regression equation.

This area of research continues to present many challenges. Given the current results and those from the long-term care sector, it appears advantageous to undertake some form of risk adjustment, even though the definitive method may not yet be best understood. Given that these HCQIs are new and so little is known about the risk adjustment process, it seems appropriate that all three type of risk adjustment be considered by researchers and policy makers. The +AIP adjustment represents the most conservative approach and may therefore be most appropriate for public reporting of HCQI results.

The assessment of quality of care, and ongoing refinement of risk adjustment methods, is ultimately intended to provide information to different audiences to assist in continuously improving the quality of care. To date, many initiatives in the US have taken place to provide additional information on the quality of LTC and home health services[6]. Similar efforts have not begun in the Canadian home care sector.

Several important issues should be addressed prior to moving towards more public reporting of these types of quality data. For example, there needs to be a discussion of the relative importance of the HCQIs. The current project made no attempt to prioritize the indicators, although clearly some would have higher clinical priority than others. To some degree, individual agencies must determine their own priorities. However, a simple system based on prevalence, severity and modifiability may be useful in determining relative importance. For example, pain is a prevalent condition in this population, can be severely debilitating for clients and is clearly modifiable. One might argue that it is of higher importance to address than declining cognitive performance, which is less prevalent and in many cases not modifiable. On the other hand, cognitive decline can have extremely serious consequences for the individual

In addition, a decision is needed regarding the calculation of the HCQIs, in particular, which pairs of assessment are to be used in the calculation of failure to improve/incidence HCQIs and what time period in between assessments is acceptable (e.g., a minimum of 90 or 120 days). There will need to be further analysis to explore the relationship between the length of stay and triggering on the HCQIs. It is anticipated that clients who have been on service longer will show important clinical differences from short-stay clients and would therefore be expected to have differential HCQI rates.

Tracking of the HCQI rates over time will be essential to determine the level of stability in the indicators. It would be useful to measure the amount of variation and to assess methods to summarize the rates over time in order to maximize their stability. For example, it may be important to report annual HCQI rates for a given organization or province if in fact the rates are found to be highly variable over shorter periods of observation. A shorter time frame may also lead to unstable rates if the number of observations is small. In this paper, a minimum of 20 observations was required and a decision would also be needed for the minimum sample size for public reporting of HCQIs.

Finally, it will be essential to assess possible methods to create summary measures across the set of HCQIs. This is not a simple task and one for which little research has been conducted to date. Previous research has pointed to the lack of correlation between measures of quality, but has also suggested that summary measures would be useful for consumers who would likely prefer less complicated information[15].

Knowledge about the home care sector in Canada remains rudimentary, and our knowledge and understanding of quality assessment in this sector is still in its infancy. The results from the current project serve to enhance the overall understanding of the issues involved in quality assessment and our ability, through the risk adjustment process, to create fair comparisons across providers.

## Conclusions

Risk adjustment of quality indicators is important to enable fair comparisons across geographic regions or across home care providers. To date, little research has examined the quality of home care services. This project, using a set of HCQIs developed by inter*RAI*, provides an important first step in assessing quality and the variable effects of different types of risk adjustment.

## Competing interests

The author(s) declare that they have no competing interests.

## Authors' contributions

DD participated in the coordination of the study, assisted in the conceptual design of the study, carried out the data analysis and took the lead in developing the manuscript. JH designed the original study, oversaw the development of the indicators, gave input into the data analysis and development of the draft manuscript. BF was the Co-Principal Investigator in the development of the indicators and provided feedback in terms of data analysis and choice of statistical methods. All authors read and approved the final manuscript.

## Pre-publication history

The pre-publication history for this paper can be accessed here:


